# Adoption and utilization of India’s eSanjeevani national telemedicine service

**DOI:** 10.1093/oodh/oqaf025

**Published:** 2025-10-07

**Authors:** Sanjay Sood, Kanhaiya Lal, Madhu Bhatia, Gagandeep Kapoor, Sumandeep Singh, Rajesh Kr Kaushish, Davinder Bisht, Vijay Kumar Sharma, Suchandrima Chakraborty, Mahesh Shete, Nitin Kumar Solanki, Aditya Naskar, Mayank Sharma, Elakeya Udhaya Subramaniyan, Shekhar Waikar, Neha Verma

**Affiliations:** Centre for Development of Advanced Computing A-34, Phase-VIII, Industrial Area, Mohali, Punjab-160071, India; Centre for Development of Advanced Computing A-34, Phase-VIII, Industrial Area, Mohali, Punjab-160071, India; Centre for Development of Advanced Computing A-34, Phase-VIII, Industrial Area, Mohali, Punjab-160071, India; Centre for Development of Advanced Computing A-34, Phase-VIII, Industrial Area, Mohali, Punjab-160071, India; Centre for Development of Advanced Computing A-34, Phase-VIII, Industrial Area, Mohali, Punjab-160071, India; Centre for Development of Advanced Computing A-34, Phase-VIII, Industrial Area, Mohali, Punjab-160071, India; Centre for Development of Advanced Computing A-34, Phase-VIII, Industrial Area, Mohali, Punjab-160071, India; Centre for Development of Advanced Computing A-34, Phase-VIII, Industrial Area, Mohali, Punjab-160071, India; Intelehealth, Office 1407, Solus Business park, Hiranandani Estate, Thane (W) Maharashtra, 400607, India; Intelehealth, Office 1407, Solus Business park, Hiranandani Estate, Thane (W) Maharashtra, 400607, India; Intelehealth, Office 1407, Solus Business park, Hiranandani Estate, Thane (W) Maharashtra, 400607, India; Intelehealth, Office 1407, Solus Business park, Hiranandani Estate, Thane (W) Maharashtra, 400607, India; Intelehealth, Office 1407, Solus Business park, Hiranandani Estate, Thane (W) Maharashtra, 400607, India; Intelehealth, Office 1407, Solus Business park, Hiranandani Estate, Thane (W) Maharashtra, 400607, India; Intelehealth, Office 1407, Solus Business park, Hiranandani Estate, Thane (W) Maharashtra, 400607, India; Intelehealth, Office 1407, Solus Business park, Hiranandani Estate, Thane (W) Maharashtra, 400607, India

**Keywords:** eSanjeevani, adoption, digital health, innovation, implementation, telemedicine

## Abstract

Telemedicine is increasingly used to expand access to primary healthcare, especially in low- and middle-income countries with workforce and infrastructure constraints. India’s eSanjeevani platform, launched in 2019, is the world’s largest government-led telemedicine initiative. The objective of this study was to analyse the adoption and utilization patterns of eSanjeevani, India’s national telemedicine service, from its national rollout in November 2019 through September 2023. We analysed aggregated programmatic data from the Centre for Development of Advanced Computing and official government sources, assessing teleconsultation volumes by platform type, state, age group, gender and diagnostic category. Over 163 million consultations were conducted across all 28 states and 8 union territories. The provider-assisted eSanjeevani Ayushman Bharat - Health and Wellness Center (AB-HWC) model accounted for >93% of usage. Utilization was highest among women and adults aged 25–45. The platform supported consultations for both acute conditions (e.g. fever, headache, diarrhoea) and chronic diseases, particularly diabetes and hypertension. The findings provide an overview of adoption and utilization trends during the platform’s expansion phase, offering a foundation for future research on patient-level outcomes, continuity of care and equity in access. The early implementation of eSanjeevani reflects the rapid scale-up of a government-led telemedicine platform in India and highlights patterns in its utilization across states, population groups and health conditions. While this study does not assess outcomes or implementation processes, it provides a foundational overview of usage trends during the programme’s expansion phase. We suggest that future research should focus on patient-level outcomes, continuity of care and equity in access, as well as the platform’s integration into broader primary healthcare delivery systems.

## INTRODUCTION

Access to essential healthcare remains a global challenge, with >4.5 billion people lacking services in 2021 and 344 million pushed into extreme poverty due to healthcare-related expenses [1]. India, despite progress in public health infrastructure, struggles to provide adequate healthcare with only 20.6 doctors and nurses per 10 000 people, and the figure falls below the World Health Organization’s recommendation of 44.5 per 10 000 [2, 3]. Even though the country has achieved a doctor–population ratio of 1:1000, 75% of doctors are urban centric leaving rural areas underserved [4–6]. March 2021 data indicate a 4.3% general physician shortage at primary health centres (PHCs) and a 79.9% specialist shortfall at community health centres, exacerbating rural healthcare disparities [7].

The Indian healthcare system also faces a dual burden of disease. While communicable, maternal and child illnesses persist, non-communicable diseases such as diabetes and hypertension are now a primary concern [8–10]. Due to the uneven distribution of healthcare providers, patients frequently bypass primary care facilities to seek treatment at secondary or tertiary centres, contributing to overcrowding and underutilisation of rural infrastructure [11]. Strengthening comprehensive primary healthcare and enabling earlier intervention are essential to ensure more equitable and effective care. In this context, telemedicine—defined as the delivery of healthcare services through telecommunications technologies—offers a promising solution for expanding access in underserved areas [12–14]. When embedded into primary care systems, telemedicine can reduce the need for physical referrals, support follow-up services and improve specialist access without requiring an in-person presence [12, 13, 15].

In response to these challenges, the Government of India launched ‘eSanjeevani’, the national telemedicine service, in November 2019 [16]. Developed by the Centre for Development of Advanced Computing (C-DAC), the platform enables remote healthcare delivery through two complementary models: (i) ‘eSanjeevani AB-HWC’, an assisted provider-to-provider service operationalized through health and wellness centres (HWCs), and (ii) ‘eSanjeevani OPD’, a direct-to-patient service for those with internet access [17–19]. The assisted model uses a hub-and-spoke architecture that connects Ayushman Arogya Mandirs (AAMs) and PHCs (spokes) to doctors at secondary or tertiary hospitals (hubs). Both services are integrated into the Ayushman Bharat Digital Mission (ABDM), allowing users to generate Ayushman Bharat Health Accounts (ABHA) and maintain longitudinal digital health records [16–18].

The COVID-19 pandemic significantly accelerated eSanjeevani’s adoption, prompting the release of national telemedicine guidelines by the National Medical Commission, Ministry of Health and Family Welfare, and NITI Aayog [20]. By September 2023, the platform had been implemented across all 28 states and 8 union territories (UTs), with >160 million consultations completed. eSanjeevani addresses a range of health needs, including acute conditions such as fever, headache, gastritis, diarrhoea and chronic diseases like diabetes and hypertension. In March 2023, the rollout of ‘eSanjeevani 2.0’ introduced enhanced features, including audio-video stability, prescription synchronization and improved integration with electronic health records. A comparative study in Haryana found that version 2.0 improved consultation quality and patient satisfaction [21].

Globally, other telemedicine platforms such as Doxy.me in the USA and national platforms in China and Europe expanded rapidly during the COVID-19 pandemic. However, these initiatives have largely operated through private or institutional systems with narrower reach. In the USA, platforms primarily catered to insured urban populations [22], while China’s services were predominantly hospital based and inpatient focussed [23, 24]. In contrast, eSanjeevani provides free outpatient consultations within a government-funded framework and is fully integrated with India’s public health system and national digital health infrastructure [25–29]. As of November 2024, eSanjeevani had facilitated >276 million teleconsultations, making it the largest documented government-led telemedicine initiative globally [28].

Despite its scale and significance, few studies have rigorously analysed the adoption and utilization of eSanjeevani using nationally representative programme data. This study addresses that gap through a descriptive quantitative analysis of aggregated programmatic data from the C-DAC, assessing adoption through trends in state-level rollout and operational infrastructure (active hubs, spokes and registered medical practitioners) and examining utilization patterns by analysing consultation volumes, patient demographics and diagnostic categories from November 2019 to September 2023. In doing so, it contributes to the growing evidence base on how government-led digital health initiatives can improve access and equity in low- and middle-income countries.

### eSanjeevani workflow

**Figure 1:**
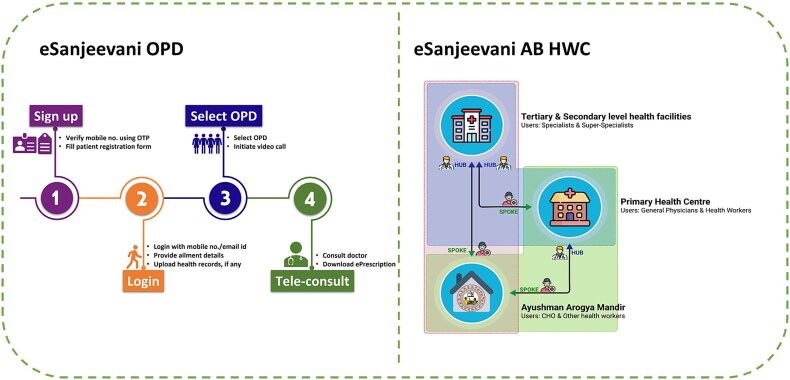
Workflow diagram of eSanjeevani OPD and eSanjeevani AB-HWC

The eSanjeevani platform operates through two distinct but complementary service delivery models: ‘eSanjeevani OPD’ and ‘eSanjeevani AB-HWC’ (Fig. 1). Together, they enable access to outpatient care for both digitally connected users and populations requiring assisted support at the primary care level.

eSanjeevani OPD is a direct-to-patient telemedicine service designed for individuals with internet-enabled devices. Patients register online, complete a short intake form, and optionally upload previous prescriptions or reports. They then select an available outpatient department and wait in a virtual queue. Once connected, a government-registered medical practitioner conducts a video consultation, reviews patient inputs and issues an e-prescription. This model is particularly suited for follow-up visits and mild conditions requiring remote care.

eSanjeevani AB-HWC follows an assisted provider-to-provider model and is deployed through a hub-and-spoke system. At the spoke site—typically a HWC, also called an Ayushman Arogya Mandir—a community health officer (CHO) conducts an initial patient assessment. If further medical input is needed, the CHO facilitates a teleconsultation with a doctor located at a hub facility such as a district or subdistrict hospital. Depending on the case, patients can be further referred to higher levels of care, forming a digitally enabled referral pathway. This model is tailored for rural and underserved populations with limited digital access or health literacy.

## MATERIALS AND METHODS

This study presents a quantitative analysis of the adoption and utilization of the eSanjeevani telemedicine platform between November 2019 and September 2023. The analysis is based on secondary programmatic data provided by the C-DAC, which manages the national backend for India’s telemedicine system. The dataset includes monthly platform-level aggregates and summary statistics on user demographics and presenting conditions.

Data on operational indicators such as the number of active hubs, spokes and registered medical practitioners (RMPs) were shared monthly and disaggregated by state and platform (eSanjeevani AB-HWC and eSanjeevani OPD). In addition, C-DAC provided summary statistics on patient gender, age group and diagnosis categories. These statistics were not based on individual consultations but were presented as aggregates for the entire platform and specific time periods. Data sources for this paper are described in Table 1.

**Table 1:** Data sources used in the study

**Data type**	**Source**	**Period**	**Disaggregation/format**
Total consultations (by platform/state)	C-DAC, Government of India	Monthly November 2019–September 2023	State, platform
Summary statistics on age and gender	C-DAC aggregated summaries	Cumulative and periodic	Age groups, gender proportions
Diagnosis category summaries	C-DAC aggregated summaries	Cumulative and periodic	Top conditions (coded summaries)
Service delivery infrastructure	C-DAC programme dashboards	Monthly	Hubs, spokes, active RMPs

Descriptive analysis and trend summaries were conducted using Microsoft Excel. Consultation volumes were grouped into calendar quarters—Q1 (January–March), Q2 (April–June), Q3 (July–September) and Q4 (October–December)—to assess patterns over time. The study focuses on identifying key trends in service expansion, demographic patterns of utilization and frequently addressed conditions. All data used were aggregate in nature, and no patient-level or identifiable health information was accessed or analysed. This methodology enables a descriptive understanding of eSanjeevani’s scale-up, its demographic reach and its service delivery patterns using the most comprehensive programmatic data available.

This methodology enables a descriptive understanding of eSanjeevani’s scale-up, its demographic reach and its service delivery patterns using the most comprehensive programmatic data available. The study was designed as non-human subjects research using only aggregate, de-identified data obtained from the C-DAC, which manages the national backend for India’s eSanjeevani telemedicine system, and as permitted under data privacy and policy guidelines of the Ministry of Health and Family Welfare. No individual-level data, personally identifiable information or health records were accessed or analysed. The researchers did not interact with any patients or providers, nor did they intervene in healthcare delivery. The analysis is entirely based on secondary programmatic data (e.g. monthly aggregates, demographic summaries, top diagnoses) already compiled for administrative purposes. There is no linkage to any other datasets that could re-identify individuals. As such, the study did not require institutional ethics review or informed consent.

## RESULTS

### Adoption of eSanjeevani—summary statistics (November 2019 to September 2023)

This section presents descriptive findings on the adoption and utilization of the eSanjeevani platform across India’s 28 states and 8 UTs. It includes analysis of teleconsultation volumes, platform-wise distribution (eSanjeevani AB-HWC and eSanjeevani OPD), demographic characteristics (age and gender), state-level patterns and the scale-up of implementation infrastructure, including hubs, spokes and registered medical practitioners.

### Number of states/UTs adopting eSanjeevani

**Figure 2:**
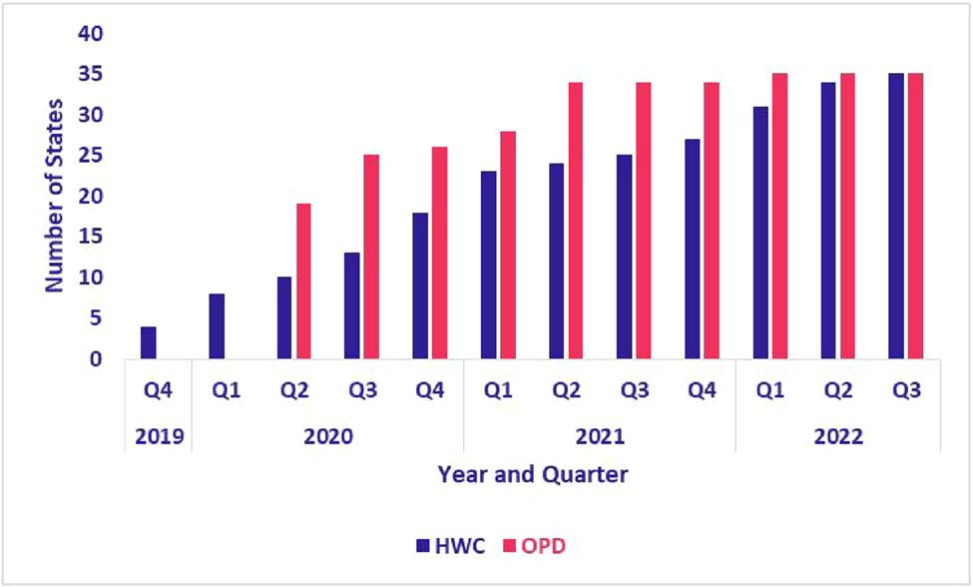
Adoption of eSanjeevani AB-HWC and OPD by states and UTs

Between November 2019 and August 2023, the adoption of the eSanjeevani platform expanded across Indian states and UTs. The assisted provider-to-provider model (eSanjeevani AB-HWC) was operational in four states and UTs at the end of 2019 and expanded to 28 states and 7 UTs by the third quarter of 2022. By 2022, the eSanjeevani platform had been adopted by 108 840 of India’s 139 389 registered AB-HWCs. State-level data on the proportions of operational AB-HWCs are provided in Table 2. The direct-to-patient model (eSanjeevani OPD), launched in early 2020, was available in 19 states and UTs by the second quarter of 2020 and reached 28 states and 7 UTs by the first quarter of 2022. As shown in Fig. 2, both models were adopted in nearly all states and UTs by August 2023.

**Table 2:** Percentage of AB-HWC utilizing eSanjeevani platform by states and UTs

**State**	**Total AB-HWC (spokes) registered (as of 2022)**	**AB-HWC operational (as of 2022)**	**Percentage of AB-HWCs operational**
Andaman and Nicobar Islands	125	33	26.4%
Andhra Pradesh	10 306	9990	96.9%
Arunachal Pradesh	309	48	15.5%
Assam	3592	2994	83.4%
Bihar	11 433	9530	83.4%
Chandigarh	59	47	79.7%
Chhattisgarh	4853	3905	80.5%
Delhi	0	0	
Goa	28	6	21.4%
Gujarat	8642	7437	86.1%
Haryana	1586	1125	70.9%
Himachal Pradesh	1578	1075	68.1%
Jammu and Kashmir	2472	1632	66.0%
Jharkhand	2691	1555	57.8%
Karnataka	9310	8065	86.6%
Kerala	1248	868	69.6%
Ladakh	268	150	56.0%
Lakshadweep	10	8	80.0%
Madhya Pradesh	10 421	8938	85.8%
Maharashtra	11 089	8447	76.2%
Manipur	262	224	85.5%
Meghalaya	563	462	82.1%
Mizoram	356	166	46.6%
Nagaland	406	28	6.9%
Odisha	5891	4863	82.5%
Puducherry	111	63	56.8%
Punjab	2686	1586	59.0%
Rajasthan	9171	5594	61.0%
Sikkim	174	155	89.1%
Tamil Nadu	8054	6432	79.9%
Telangana	5973	5895	98.7%
The DNH and DD	97	94	96.9%
Tripura	710	466	65.6%
Uttar Pradesh	16 037	10 117	63.1%
Uttarakhand	1380	896	64.9%
West Bengal	7498	5946	79.3%
India	139 389	108 840	78.1%

**Figure 3:**
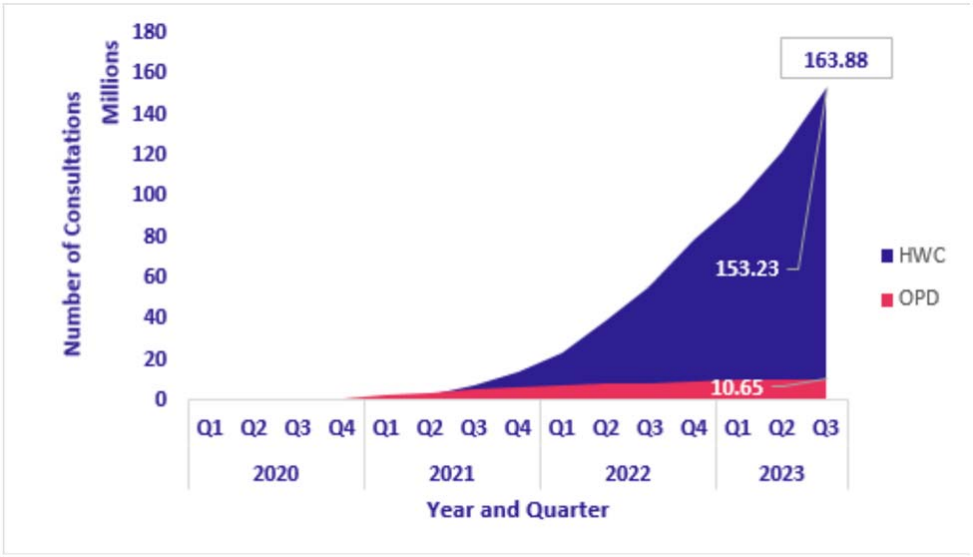
Cumulative teleconsultations completed (eSanjeevani AB-HWC and OPD)

Figure 3 shows the cumulative number of teleconsultations conducted via eSanjeevani AB-HWC and eSanjeevani OPD from January 2020 to September 2023. Utilization increased steadily over time, with a sharp rise in eSanjeevani OPD consultations between Q2 2020 and Q3 2021, coinciding with the COVID-19 waves. In contrast, eSanjeevani AB-HWC displayed a more gradual, linear growth pattern throughout the period.

**Figure 4:**
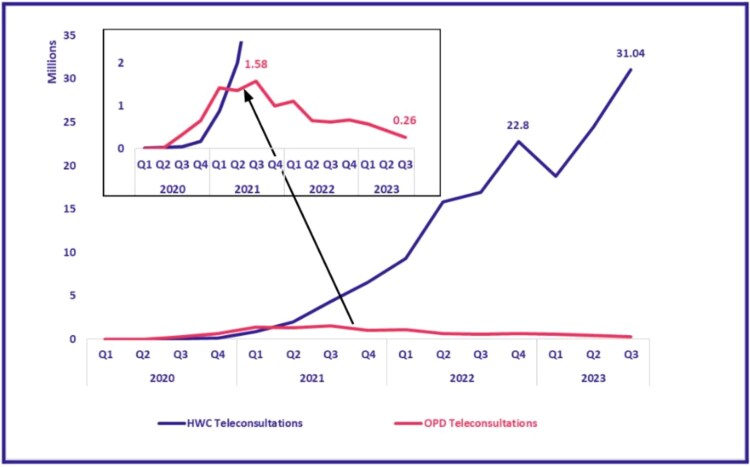
Quarter-wise teleconsultations completed (eSanjeevani AB-HWC and OPD)

Figure 4 presents quarter-wise growth rates for each platform. eSanjeevani AB-HWC showed consistent quarterly increases, ranging from 7% to 398%, reflecting its phased integration into primary healthcare. eSanjeevani OPD experienced more volatile growth, with a 443% increase in 2021 alone, corresponding with pandemic-related demand surges. The two platforms exhibited distinct utilization patterns: steady uptake in the assisted model and event-driven peaks in the direct-to-patient model.

### State-wise distribution

**Figure 5:**
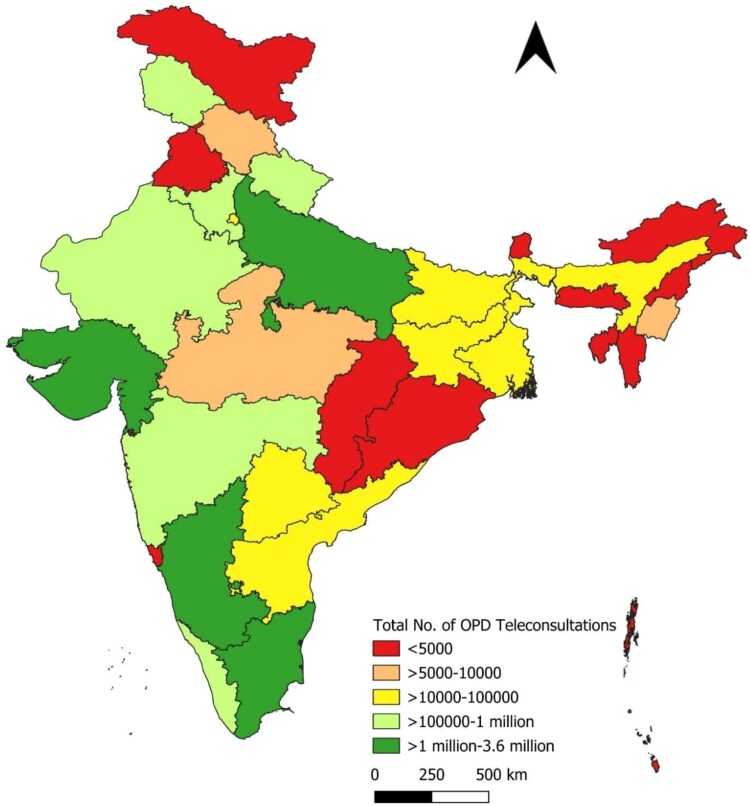
State-wise distribution of eSanjeevani AB-HWC teleconsultations

**Figure 6:**
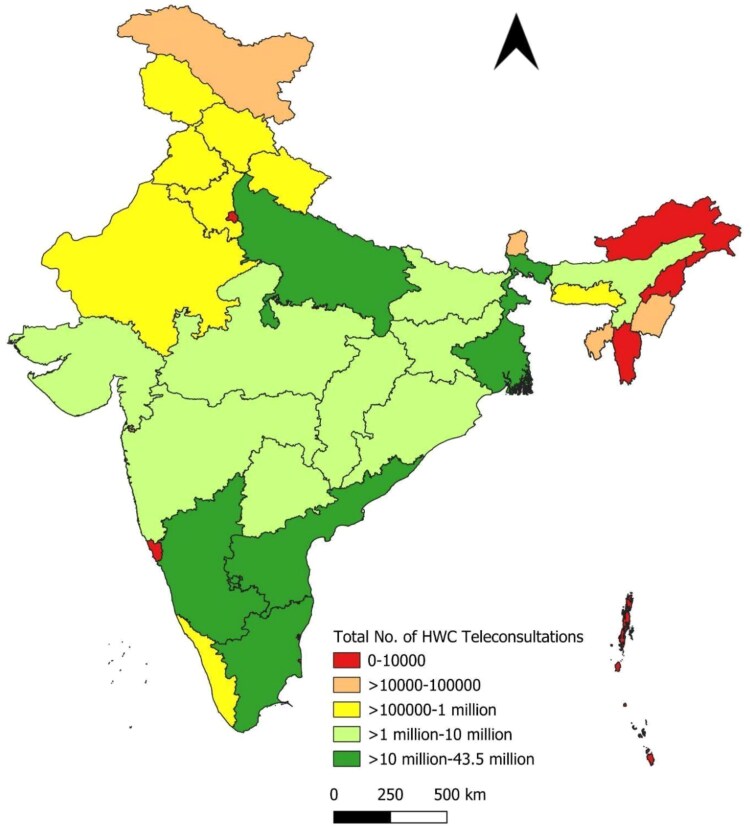
State-wise distribution of eSanjeevani OPD teleconsultations

**Figure 7:**
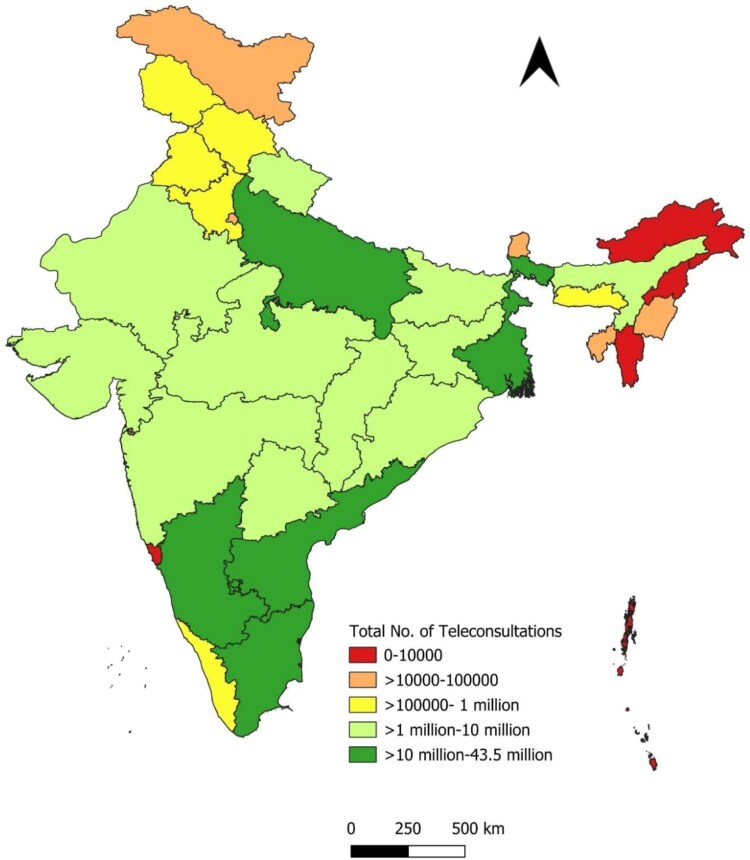
State-wise distribution of total teleconsultations

Figures 5, 6 and 7 and Table 3 present the distribution of teleconsultations across states from January 2020 to September 2023. A small number of states accounted for a disproportionately large share of total consultations. The top five contributors—Tamil Nadu, Uttar Pradesh, Madhya Pradesh, Karnataka and Andhra Pradesh—collectively accounted for ~70.6% of all teleconsultations conducted through eSanjeevani. These states also contributed the highest volumes across both platform models. This distribution likely reflects a combination of factors, including population size, service readiness and programmatic rollout timelines.

**Table 3:** Top 5 states with the highest teleconsultations (January 2020–September 2023)

**S. no.**	**No. of total teleconsultations**	**No. of eSanjeevani AB-HWC teleconsultations**	**No. of eSanjeevani OPD teleconsultations**
1	Andhra Pradesh—43 499 765	Andhra Pradesh—43 465 307	Karnataka—3 678 203
2	West Bengal—22 868 786	West Bengal—22 856 605	Uttar Pradesh—1 830 597
3	Tamil Nadu—21 531 609	Tamil Nadu—19 784 090	Tamil Nadu—1 747 519
4	Karnataka—14 754 405	Karnataka—11 076 202	Gujarat—1 014 158
5	Uttar Pradesh—12 267 662	Uttar Pradesh—10 437 065	Uttarakhand—848 230

### Expansion of eSanjeevani hubs and spokes

**Figure 8:**
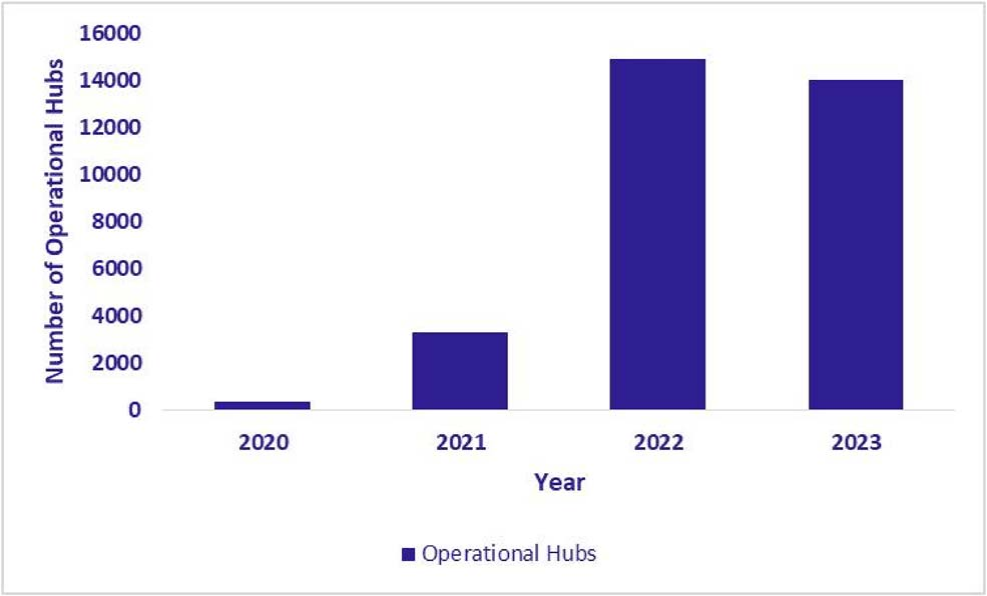
Growth in operational hubs (January 2020–September 2023)

**Figure 9:**
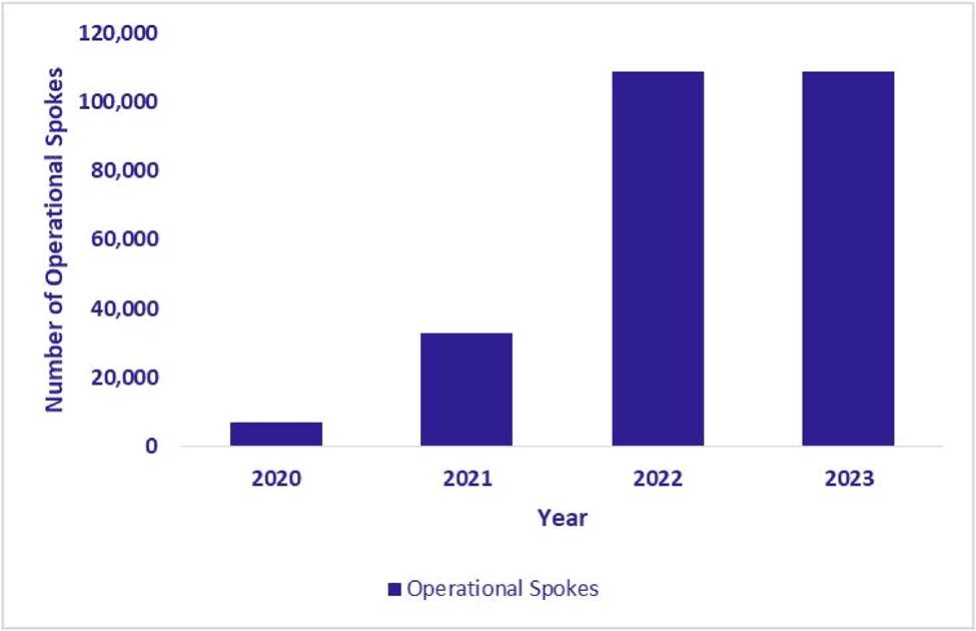
Growth in operational spokes (January 2020–September 2023)

**Figure 10:**
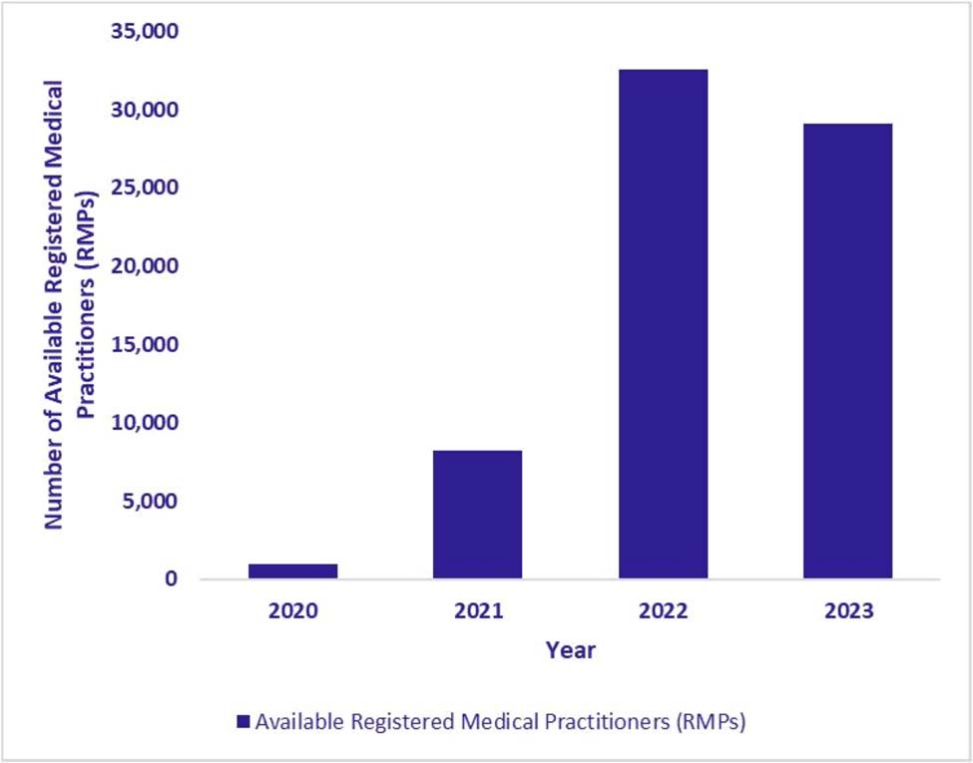
Number of available registered medical practitioners (RMPs) (January 2020–September 2023)

The expansion of the eSanjeevani platform was directly accompanied by a corresponding increase in its service delivery infrastructure. As illustrated in Figs. 8, 9 and 10, the number of operational hubs (secondary or tertiary facilities providing remote consultations) increased from 397 in 2020 to 14 036 by September 2023. The number of spokes, which primarily include HWCs implementing eSanjeevani AB-HWC, grew from 6868 in 2020 to 108 610 during the same period. In addition, the number of RMPs who had provided at least one consultation (in a year) via the platform reached 29 114 by September 2023. These figures reflect a substantial expansion of telemedicine infrastructure across primary and higher-level facilities.

### Telemedicine utilization by gender

**Figure 11:**
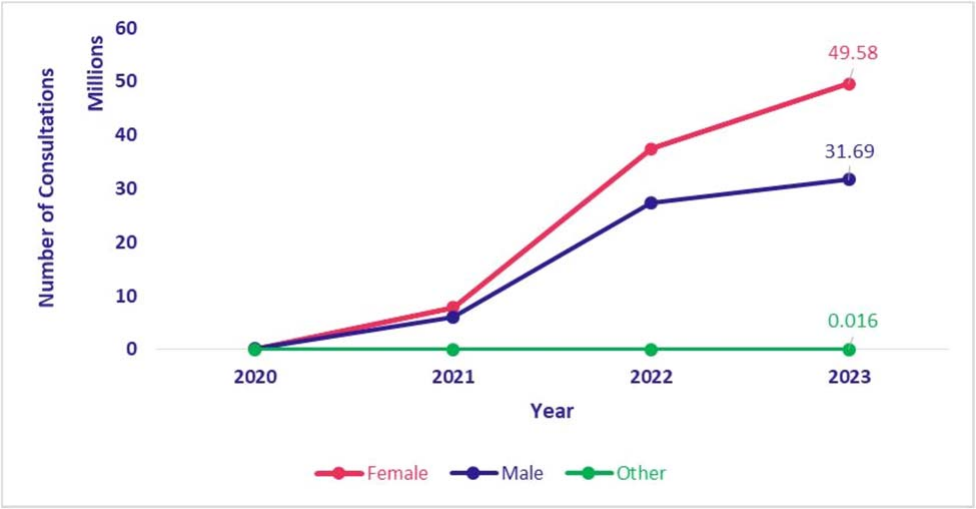
eSanjeevani OPD utilization by gender (April 2020–September 2023)

**Figure 12:**
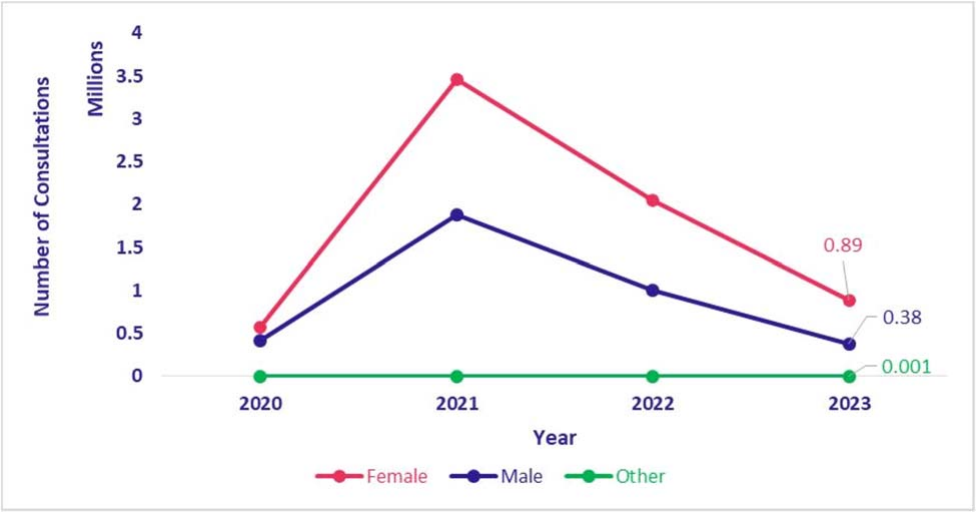
eSanjeevani AB-HWC utilization by gender (January 2020–September 2023)

Figures 11 and 12 present the distribution of teleconsultations conducted through eSanjeevani AB-HWC and eSanjeevani OPD from 2020 to 2023, disaggregated by gender. A notable trend is the consistently higher utilization of services by women. In eSanjeevani AB-HWC, female clients accounted for 59.8% (155 903 consultations) compared to 40.2% (104 720 consultations) by male clients in 2020. In subsequent years, the share of female consultations remained stable, ranging between 56.9% and 57.7%. In eSanjeevani OPD, women’s share of teleconsultations increased from 57.6% (567 827 consultations) in 2020 to 70.1% (885 811 consultations) in 2023. During the same period, men’s share declined from 42.4% (417 628 consultations) to 29.8% (376 947 consultations), indicating an increase in proportion of teleconsultations for women.

### Telemedicine utilization by age

**Figure 13:**
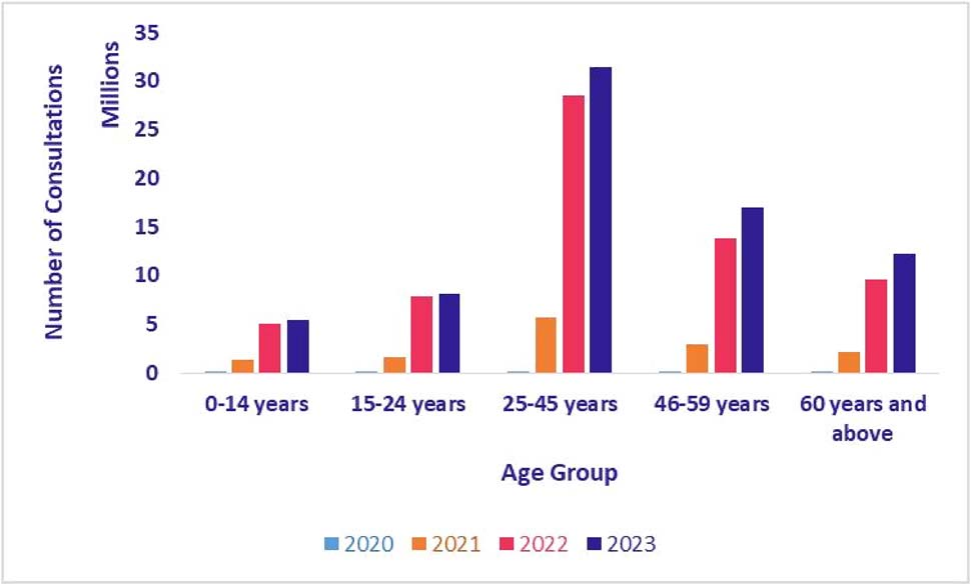
Age-wise teleconsultation—eSanjeevani AB-HWC (April 2020–September 2023)

**Figure 14:**
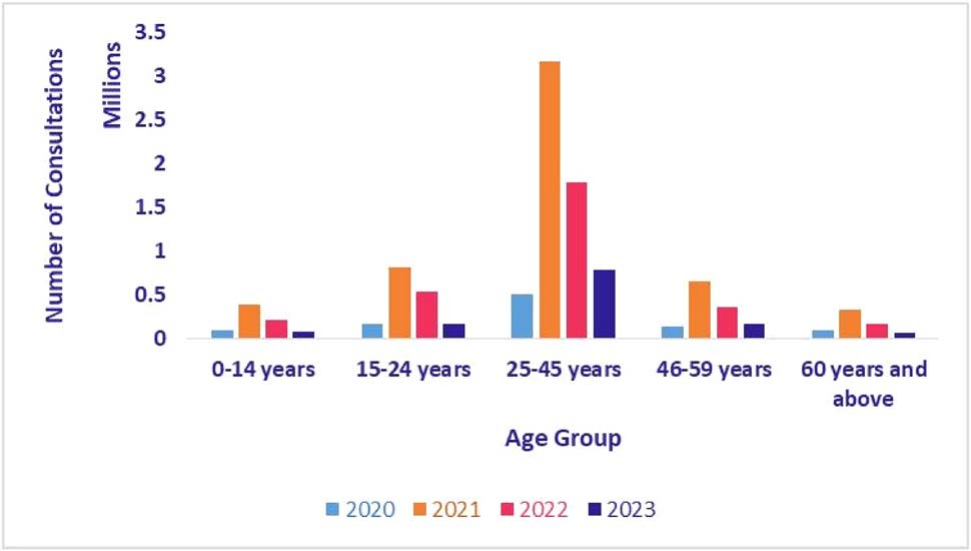
Age-wise teleconsultation—eSanjeevani OPD (April 2020–September 2023)

Figures 13 and 14 show the age distribution of teleconsultations on eSanjeevani AB-HWC and eSanjeevani OPD from 2020 to 2023. In both platforms, users aged 25–45 consistently made up the largest share of consultations. In eSanjeevani AB-HWC, this group accounted for 36.7% of consultations in 2020 and rose to >42% by 2023. In eSanjeevani OPD, the same group represented more than half of all consultations each year, increasing from 50.7% in 2020 to 62.2% in 2023. Children (0–14 years) and older adults (60+ years) used the platform less, though their usage also increased in 2021.

### Telemedicine utilization by health conditions

Table 4 presents the top 10 diagnoses in eSanjeevani OPD, dominated by acute conditions such as common cold, fever and headache. In cases where multiple symptoms coexisted (e.g. diarrhoea, vomiting and fever), each diagnosis was recorded as a separate entry. Table 5 presents the most frequently reported health conditions in eSanjeevani AB-HWC across versions 1.0 and 2.0. While common cold, fever and headache remained among the top consultations, version 2.0 shows increased usage for chronic disease management, particularly for follow-up for diabetes and hypertension (The platform did not employ standardized diagnostic coding systems (e.g. ICD-10 or SNOMED CT), and the aggregated data do not distinguish between single and coexisting diagnoses.).

**Table 4:** Top 10 diagnoses with highest teleconsultations, eSanjeevani OPD (April 2020–September 2023)

**eSanjeevani OPD**		
**S. no.**	**Diagnosis name**	**Number of teleconsultations**
1	Common cold	1 268 185
2	Fever	1 060 580
3	Headache	6 53 923
4	Backache	3 15 489
5	Abdominal pain	2 29 671
6	Myalgia	1 80 153
7	Gastritis	1 69 973
8	Vomiting	1 21 366
9	Joint pain	83 414
10	Toothache	79 488

**Table 5:** Top 10 diagnoses with highest teleconsultations, eSanjeevani AB-HWC 1.0 and 2.0 (January 2020–September 2023)

	**eSanjeevani AB-HWC 1.0 (January 2020 to February 2023)**		**eSanjeevani AB-HWC 2.0 (March 2023–September 2023)**	
	**Diagnosis**	**No. of teleconsultations**	**Diagnosis Name**	**No. of teleconsultations**
1	Common cold	7 720 104	Common cold	4 231 930
2	Fever	6 454 433	Fever	3 640 890
3	Headache	5 356 335	Headache	2 857 760
4	Gastritis	2 590 654	Gastritis	2 146 458
5	Backache	2 048 108	Joint pain	1 927 494
6	Generalized body pain	19 163 44	Abdominal pain	5 43 683
7	Diarrhoea	1 715 612	Weakness	3 87 095
8	Abdominal pain	1 659 784	Diabetes mellitus	3 27 491
9	Vomiting	1 520 477	Vomiting	2 79 709
10	Joint pain	1 487 660	Hypertension	2 71 253

A total of 327 491 consultations related to follow-up for diabetes mellitus were recorded. These were primarily follow-up consultations, with diagnoses typically established during in-person visits at public health facilities using confirmatory tests such as HbA1c. eSanjeevani AB-HWC was used to monitor disease progression, manage medication adherence or address complications. Although the data suggest continuity of care, repeat visit counts could not be confirmed, as beneficiary-level data were not available (As the analysis is based on summary statistics provided by C-DAC, individual consultation records and clinical histories were not available for further disaggregation.).

### Average time of teleconsultation

As per figures reported by the C-DAC, the national average duration of an eSanjeevani teleconsultation was ~1 min and 15 s as of September 2023. This measure reflects only the active interaction time between the doctor and the patient on the telemedicine platform. It does not capture waiting time before the consultation or the total turnaround time for a case, which may also include reviewing patient records, diagnostic reports or completing documentation. Disaggregated figures by state, platform type or case category were not available.

## DISCUSSION

This study examines national trends from the early implementation period of eSanjeevani, India’s public-sector telemedicine platform. Between January 2020 and September 2023, the platform delivered >163 million consultations, of which >93% of consultations were delivered through the provider-assisted eSanjeevani AB-HWC model, indicating its central role in the platform’s overall utilization. Adoption extended across all 28 states and 8 UTs, with a few early-implementing states accounting for a substantial share of consultations. Women constituted the majority of users across both platforms, and the 25–45-year age group accounted for the highest share of consultations. While acute conditions such as fever and common cold remained prevalent (Table 4), use for chronic conditions, particularly diabetes and hypertension, also increased in version 2.0 of eSanjeevani AB-HWC, indicating evolving patterns in service use over time.

The rise and later decline in eSanjeevani OPD usage reflect heightened demand for remote consultations during the COVID-19 pandemic [19], followed by a return to in-person services. In contrast, eSanjeevani AB-HWC continued to grow steadily, aligning with its phased integration into HWCs [16]. Increased use for chronic disease management, like in the case of diabetes follow-ups, coincided with the availability of version 2.0 [21] and its compatibility with point-of-care devices. However, due to the absence of individual-level identifiers, it is not possible to determine whether these consultations represent first-time visits for symptom presentation or follow-up care after an initial diagnosis.

During the COVID-19 pandemic, several Indian states, notably Tamil Nadu, Andhra Pradesh and Karnataka, were among the states with early high uptake of eSanjeevani telemedicine, showing large numbers of consultations. These trends coincide with efforts in many states to train health personnel, deploy telemedicine platforms and expand digital health ecosystems. [29–31]. Supportive policy frameworks have been essential to eSanjeevani’s early expansion. The release of the National Telemedicine Guidelines [20], integration with Ayushman Bharat Digital Mission [16, 18], and government prioritization during the pandemic created enabling conditions for programme growth. The introduction of eSanjeevani 2.0 in 2023 further strengthened the platform by improving audio-video stability, record synchronization and point-of-care integration [21]. Awareness campaigns and state-level engagement also contributed to user uptake.

The higher utilization among women is notable, given persistent digital access and mobility constraints [32]. It may reflect how the assisted model (delivered through CHOs at HWCs) reduces barriers to access. These patterns are observed in aggregate trends and further studies are needed to explore the mechanisms driving gendered engagement with telemedicine, including perceptions of care, digital literacy and household decision-making. The data analysed in this paper are unable to capture how gender intersects with rurality, caste, socioeconomic status or geography in shaping access. Prior evidence suggests that digital gender gaps, smartphone restrictions and limited mobility may still prevent marginalized women from independently accessing telemedicine, particularly in northern states with stricter norms around female autonomy [32]. Future studies should explore intersectional barriers to telemedicine uptake by combining user-level data with qualitative insights, disaggregating patterns by gender, rural/urban status and state-specific norms. Such analysis would help identify subgroups that are underrepresented despite high overall usage, guiding more equitable service design.

Despite its reach, eSanjeevani faces several challenges common to digital health interventions. Teleconsultation uptake is shaped by digital infrastructure, smartphone ownership and internet connectivity—factors that remain limited in rural and low-income communities. Only 38% of Indian households are considered digitally literate, with wide rural–urban and gender gaps [32]. The eSanjeevani AB-HWC model helps bridge these gaps through CHO-assisted consultations; however, barriers remain, including long waiting times [33], platform navigation issues and limited availability of medical officers for real-time consultations. Gender norms in some areas—such as restrictions on women’s mobile phone usage—also impact access [32]. Some states have piloted mitigation strategies, such as deploying dedicated telemedicine practitioners or establishing public–private partnerships to supplement provider availability [34]. These models merit further evaluation to assess scalability and sustainability.

This analysis is based on programme-level summary data shared by the C-DAC, which manages the backend infrastructure for eSanjeevani [15]. Individual-level data were not accessible, and for this period of assessment the platform does not track beneficiaries through unique identifiers like ABHA (The Ayushman Bharat Health Account (ABHA) is a unique 14-digit health ID issued under India’s Ayushman Bharat Digital Mission (ABDM). It enables individuals to link and access their health records across public and private health systems through a secure, consent-based digital platform.) accurately. As a result, it was not possible to determine the number of unique users, measure per capita coverage, or distinguish between new and repeat consultations. This limits the ability to assess care continuity or patient-level outcomes. However, secondary evidence from a detailed state-level implementation study in Jharkhand provides further insight into user engagement and service quality on the eSanjeevani platform. Between May 2021 and March 2022, the telemedicine service recorded a 1000-fold increase in utilization, growing from 50 to >50 000 consultations per month. Treatment compliance was reported at 96.5% among those who received a prescription or medical advice, and 88% of patients were able to access medicines at their HWC. Notably, 31% of surveyed clients reported using the platform more than once, indicating early signs of repeat engagement and platform stickiness. Patient-reported outcomes also indicated a high rate of self-reported recovery: 60% fully recovered and 25% partially recovered from their health problems following a teleconsultation. These findings from Jharkhand, though subnational, reinforce the potential of eSanjeevani to deliver consistent, acceptable and reusable outpatient care in low-resource settings when embedded within primary healthcare infrastructure. [35]

### Limitations

This study has several important limitations that should be acknowledged when interpreting the findings. First, our analysis is based exclusively on secondary, aggregate and de-identified data provided by the C-DAC. The data were compiled primarily for administrative and programme monitoring purposes, not for research, which constrains what can be inferred about individual behaviour, quality of care or service delivery nuances.

Second, the aggregated nature of the data means that we lack consultation-level records, and particularly state-wise gender distributions, or the ability to link gender with specific presenting diagnoses. As a result, we cannot determine whether observed gender differentials in usage are driven by differences in health condition burden, access or health-seeking behaviour.

Third, we were unable to analyse the duration of consultations by condition (acute vs. chronic) or assess other time-related metrics such as waiting times or provider turnaround. Such metrics could be informative about quality, intensity and patient satisfaction but were not available in our data sources.

Fourth, data on the infrastructure and resource differences across states (e.g. the number and training levels of CHOs, readiness of digital systems or internet connectivity) are not available in the dataset. Therefore, statements about states with stronger digital infrastructure and faster uptake rely on inference rather than direct measurement.

Finally, because the data cover national and state aggregates, we cannot assess causality—whether stronger infrastructure ‘caused’ higher adoption or whether other factors (political leadership, funding, population density, etc.) played major roles. There may also be reporting delays, differences in how states define or record consultations, and variation in data completeness.

The reliance on aggregate, non–individual-level data may bias interpretation towards overestimating acute conditions, which are more likely to prompt one-time consultations, while underrepresenting continuity of care for chronic illnesses. Without tracking repeat visits or patient identifiers, it is also not possible to assess user retention or treatment completion. As a result, trends may reflect service contact volume rather than sustained healthcare engagement. Furthermore, the system does not use standardized diagnostic coding (e.g. ICD-10 or SNOMED CT), and it is unclear whether coexisting symptoms (e.g. fever, diarrhoea, vomiting) were recorded independently or as a composite. Facility classification is also limited as many different types of facilities (e.g. AAMs, PHCs, SCs) were grouped under HWCs in the available data. Only one state-level qualitative report was available to triangulate findings, limiting the interpretability of user experience and provider behaviour.

These limitations reflect the current design of national-level monitoring systems rather than data quality concerns. As the programme integrates with ABDM and ABHA-linked infrastructure, future evaluations should leverage individual identifiers to assess follow-up behaviour, treatment outcomes and service delivery equity. In the interim, facility-based studies and surveys can complement aggregate monitoring with more detailed evidence.

While this paper focusses on teleconsultation volumes and trends, it does not assess how eSanjeevani integrates with downstream elements of primary care—such as access to medicines, diagnostics or in-person referrals. Understanding whether patients are able to complete treatment plans initiated remotely remains a critical gap. Future evaluations should examine how telemedicine platforms interface with supply chains, referral systems and physical follow-up services to ensure continuity and effectiveness of care beyond the consultation itself.

## CONCLUSION

eSanjeevani represents a significant government-led initiative to expand access to outpatient care through telemedicine in India. Its early implementation shows strong uptake across all states and UTs, with the provider-assisted eSanjeevani AB-HWC model emerging as the dominant mode of delivery. The platform has demonstrated potential to support both acute and chronic care management, particularly in rural settings where physical access to doctors is limited.

However, realizing its full potential will depend on addressing current system limitations, including the lack of individual-level tracking, diagnostic standardization and real-time analytics. As eSanjeevani continues to evolve, its integration into comprehensive primary care beyond minor ailments will be essential. Ensuring digital equity, provider availability and robust data systems will be key to improving both reach and quality of care. With continued investment in monitoring, evaluation and infrastructure, eSanjeevani offers a replicable model for scaled public-sector telemedicine in low- and middle-income countries.

## Data Availability

The data underlying this article cannot be shared publicly due to restrictions imposed by the Government of India on patient-level and provider-level health records. In compliance with the Digital Personal Data Protection (DPDP) Act, 2023, health data are considered sensitive personal data and cannot be shared with non-governmental entities without explicit authorization, in order to protect the privacy of individuals and prevent re-identification. The dataset used for this analysis was provided in aggregate and de-identified form by the Centre for Development of Advanced Computing (C-DAC) under the Ministry of Health and Family Welfare, solely for research purposes. The data may be shared in aggregated form on reasonable request to the corresponding author, subject to necessary approvals from the relevant authorities.
